# The Blast Resistance Gene *Pi54of* Cloned from *Oryza officinalis* Interacts with *Avr-Pi54* through Its Novel Non-LRR Domains

**DOI:** 10.1371/journal.pone.0104840

**Published:** 2014-08-11

**Authors:** Navadagi B. Devanna, Joshitha Vijayan, Tilak R. Sharma

**Affiliations:** National Research Centre on Plant Biotechnology, Indian Agricultural Research Institute, New Delhi, India; International Rice Research Institute, Philippines

## Abstract

The dominant rice blast resistance gene *Pi54* cloned by map-based cloning approach from indica rice cultivar Tetep confers broad spectrum resistance to *Magnaporthe oryzae*. In this investigation, an orthologue of *Pi54* designated as *Pi54of* was cloned from *Oryza officinalis* conferring high degree of resistance to *M. oryzae* and is functionally validated. We have also characterized the Pi54of protein and demonstrate its interaction with AVR-Pi54 protein. The *Pi54of* encoded ∼43 kDa small and unique cytoplasmic LRR family of disease resistance protein having unique Zinc finger domain overlapped with the leucine rich repeat regions. *Pi54of* showed *Magnaporthe*-induced expression. The phylogenetic and western blot analysis confirmed orthologous nature of *Pi54* and *Pi54of* genes, whereas the identity of protein was confirmed through MALDI-TOF analysis. The *in silico* analysis showed that Pi54of is structurally more stable than other cloned Pi54 proteins. The molecular docking revealed that Pi54of protein interacts with AVR-Pi54 through novel non-LRR domains such as STI1 and RhoGEF. The STI1 and GEF domains which interact with AVR-Pi54 are also components of rice defensome complex. The Pi54of protein showed differential domain specificity while interacting with the AVR protein. Functional complementation revealed that *Pi54of* transferred in two rice lines belonging to indica and japonica background imparts enhanced resistance against three highly virulent strains of *M. oryzae*. In this study, for the first time, we demonstrated that a rice blast resistance gene *Pi54of* cloned from wild species of rice provides high degree of resistance to *M. oryzae* and might display different molecular mechanism involved in AVRPi54-Pi54of interaction.

## Introduction

Rice is the staple food for larger part of the world population. The major biotic stress of rice which is considered as one of the most damaging diseases worldwide is rice blast caused by hemibiotrophic fungi *Magnaporthe oryzae*. In India alone, the total loss due to rice blast during 1960–1961 was 2, 65,000 T amounting to about 0.8% of total rice production. [1]. However, blast under severe epiphytic conditions may result between 70–90% loss in isolated fields/localities. Among the other control measures, use of resistance (*R*) -genes with overlapping resistance spectra is most viable approach [2]. *M. oryzae* is highly adaptive and infects rice plants at all developmental stages [3]. The adaptive nature of *M. oryzae* is mainly because of breakdown in host resistance due to mutations in the pathogen leading to instability of avirulence (*AVR*) genes [4]. The fungus can overcome even major *R* gene-mediated resistance with the mutation in the corresponding *AVR* gene limiting the life span of major *R-*genes [5], [6]. This necessitates time bound release of resistant varieties for effective management of rice blast. In view of this, continued identification and cloning of new effective genes and their alleles are critical for *R*-gene mediated management of rice blast disease.

The wild species of rice harbour resistance genes that remain largely unexploited [7]. It is important to clone the *R-*gene (s) to establish their functional character for their effective and meaningful utilization. The resistance genes against bacterial blight from *O. longistaminata* (*Xa21*) and *O. officinalis*, brown plant hopper transmitting grassy stunt virus from *O. nivara* and white backed plant hopper from *O. officinalis* have been transferred in many varieties [8]. Wild species of rice such as *O. rufipogon* and *O. officinalis* have been reported to be a potential source for blast resistance genes [9]. In case of rice, many genes, such as *Pi9* from *O. minuta*, *Pi-40(t)* from *O. australiensis*, *Pirf2-1*(t) and *Pid3-A4* from *O. rufipogon*, *Pi54rh* from *O. rhizomatis* have been shown to be effective against blast disease [10]. Allele mining of the gene from the wild and cultivated species of rice not only aid in identification of genetic variation and to understand evolution, but also serves as a tool for identification of the better alleles. In rice, a strong rice blast resistance *Pi-ta(+)* allele has been isolated from wild rice *O. rufipogon* [11].

During the long co-evolution with pathogens, the plants have developed sophisticated mechanisms to perceive and defend against a pathogen’s attack [12]. The plant innate immunity is classified into basal and *R*-gene-mediated resistance [13]. The first line of plant defense against invading pathogens is activated by membrane proteins called pattern recognition receptors (PRRs), which recognize the conserved pathogen associated molecular patterns (PAMPs). Some plant pathogens bypass this PAMP-triggered immunity (PTI) through effector molecules and trigger disease reaction by manipulating key components of the plant defense [14]. To overcome this infection, plants do have a second layer of defense called effector-triggered immunity (ETI) which relies on the specific recognition of certain pathogen-derived effector molecules called avirulence (AVR) proteins by the plant R- proteins. All the *R-*genes cloned till date from different plant species have been divided into five classes based on their protein structure [15]. The largest class of these *R*-genes encode NBS-LRR family of proteins. Pathogen AVR proteins are detected by R- proteins either through direct or indirect interaction. Direct R–AVR interaction has been reported for nine R- proteins: the first one being Pi-ta, followed by RRS1-R, N, L5/L6, M, RPP1, Pik/km/kp/ks/kh, RGA5, and Pb1 [16].

The recent developments in the field of molecular biology and genomic resources, besides the genetic tractability and economic significance of blast disease have made the *rice–Magnaporthe* interaction as a model system for the analysis of plant–pathogen relationships. Race specific *R*-gene-mediated resistance to *M. oryzae* closely follows the classical gene-for-gene relationship [17]. The isolation and characterization of *R*-genes will help to unravel varied molecular mechanisms underlying the interaction between host and the pathogen. Till-date, around 100 *R*-genes have been identified and many of them mapped on the rice genome and 23 of them (*Pib*, *Pita*, *Pi54*, *Pi-9*, *Pid2, Pi*2, *Piz-t*, *Pi-36*, *Pi-37*, *Pikm*, *Pi5*, *Pid3*, *pi21*, *Pit*, *Pb1*, *Pish*, *Pi-k*, *Pik-p*, *Pia, NLS1,Pi25, Pi54rh* and *Pid3-A4*) have been cloned and characterized [8]. Interestingly, 19 of them are NBS-LRR genes, while, *Pi54* and *Pi54rh* predicted to have rudimentary NBS domain and small LRR domains [18]; *Pi-d2* and *Pi21* code for non NBS-LRR proteins [16].

Among the cloned rice blast *R*-genes, only the functional characterization of protein encoded by *Pita* and its effector has been performed in detail [19]. The yeast two hybrid experiments showed that the rice blast resistance gene *RGA5* product interact directly with its AVR protein through non-LRR domain and *Pb1* gene product with WRKY45 through its CC domain [16], [20]. However, details of molecular mechanisms linking R–AVR interaction with the activation of downstream disease resistance signalling pathways are largely unknown. These R- proteins encoded by rice blast resistance genes are very diverse in their size, AVR recognition specificity and resistance mechanism. So, *in vitro* expression systems are indispensable for expression, purification and structural and functional characterization of proteins involved in plant disease resistance [21].

Allele mining entails PCR-based amplification and sequencing of the different versions of the genes found in the germplasm. Variation in gene sequence is then correlated with the phenotypic trait or performance of the accession to enable the identification of favourable alleles for future experiments. The cloned dominant blast resistance gene *Pi54* has been effective against a selective set of rice blast pathogen isolates from different parts of India [22], [23]. The wild rice line *O. officinalis* is highly resistant to virulent strains of *M. oryzae* like PLP-1, the most predominant isolate widely distributed in the North-western Himalayan region of India [24] and Mo ei-11 from Hazaribagh region of eastern India. Therefore, allele mining for *Pi54* gene in this wild rice might reveal more effective resistance orthologue of *Pi54* gene. We report in this study cloning of an orthologue of rice blast resistance gene *Pi54* from wild rice *O. officinalis* and its molecular and functional characterization.

## Materials and Methods

### Plant material, fungal strains and oligos

The wild rice *O. officinalis* (nrcpb004) is being maintained under controlled conditions at this institute. Seeds of indica rice lines IET16310 and HR12 were obtained from the Division of Genetics of the Indian Agricultural Research Institute, New Delhi. The japonica rice line Taipei 309 (TP309) was available with the authors. A highly virulent isolate of *M. oryzae*, Mo ei-11 was obtained from the Central Rainfed and Upland Rice Research Station, Hazaribagh, Jharkhand, India and maintained in the Centre’s stock culture was used for phenotypic analysis of *O. officinalis*. The fungal strains RML21 and MG-79 were obtained from the Department of Agricultural Biotechnology, CSK Himachal Pradesh Agricultural University, Palampur, Himachal Pradesh, India and 6-Sikkim was obtained from the Division of Plant Pathology, Indian Agricultural Research Institute, New Delhi, India. The list of oligos used in this study is given in Table S1 in File S1.

### Phenotyping with *Magnaporthe oryzae*

Phenotyping of fifteen-day-old young seedlings of rice variety HR12 and vegetatively propagated plantlets (two leaf stage) of wild rice *O. officinalis* were inoculated with *M. oryzae* spore suspension (1×105 spores/ml) as described by Sharma et al. [22]. The experiment was carried out under controlled growth conditions at 25±1°C and 90% RH for initial 24 h in dark and then shifted to 16/8-h light/dark regime. The disease reaction observed in each rice line was recorded after 7 days post inoculation using 0–5 disease rating scale [25].

### Cloning of full length *Pi54of* gene

PCR amplification of *Pi54of* orthologue was performed with primers designed from the BAC clone sequence (AC145349.1) of the *O. sativa* cv. Nipponbare (IRGSP 2005) corresponding to the location of *Pi54* gene region. Amplified PCR product was gel purified and cloned in pGEMT-E vector (Promega) and sequenced (Xcelris Labs Ltd, India). To clone the complete functional *Pi54of* gene, inverse PCR (iPCR) was carried out using *Pi54of* specific primers following standard protocol [26]. For this, self ligated product of *Eco*RI digested *O. officinalis* genomic DNA was used as template. The iPCR product was cloned, sequenced and the sequence reads obtained were assembled using Phred/Pharp/Consed Software Package (http://www.phrap.org/). Further, 5′ Rapid Amplification of cDNA Ends (5′RACE) PCR was conducted by First Choice RLM-RACE kit (Ambion, Austin, Texas, USA), following the manufacturer’s instructions. The total RNA isolated from *O. officinalis* plants at 72 hours post inoculation (hpi) with *M. oryzae* was used as template. The PCR product was cloned in pGEMT-Easy vector (Promega) and sequenced.

### Real-time PCR analysis

Total RNA was isolated from HR12 and *O. officinalis* plantlets pre- and post- inoculation with the *M. oryzae* isolate Mo-ei-11 and mock (0.25% gelatine) control. Total RNA was extracted from leaf tissues after 12, 24, 48, 72 and 96 h of inoculation using the Spectrum Plant Total RNA Kit (Sigma). The qRT-PCR analysis was conducted using *Pi54of* ORF rice elongation factor-1 alpha (EF-1α) gene specific primers in a reaction mix of KAPA SYBR FAST qRT-PCR Master mix (Kapa Biosystems, Inc. USA) according to the manufacturer’s protocol and reaction was run using Light Cycler 480 II PCR system (Roche).

### Structural and comparative analysis of *Pi54of*

DNA sequence similarity search was done using BLASTn (blast.ncbi.nlm.nih.gov) and the nucleotide sequence comparison was done using BioEdit Ver. 7.0.9 (www.mbio.ncsu.edu). Structural annotation was done with FGENESH (http://linux1.softberry.com/) and PLACE (http://www.dna.affrc.go.jp/PLACE/) softwares. The amino acid sequence of *R-*gene products was downloaded from NCBI and was used for the phylogenetic tree construction based on the progressive multiple sequence alignment using the Neighbor-Joining method with 1000 replicates in the MEGA6 program (http://www.ncbi.nlm.nih.gov/) [27].

### Analysis of physico-chemical properties of the proteins of *Pi54* orthologues

The physico-chemical properties of the protein sequences of *Pi54* orthologues were calculated by using Protparam software (http://web.expasy.org/protparam/). The secondary structures and number of bonds were calculated by using protein visualization software RasMol (http://www.openrasmol.org/doc/). The COILS server was used to predict coiled coil domain [28]. GlobPlot-2 analysis was performed to identify the presence of Globular domains and disorder regions (http://globplot.embl.de/). Various domains were predicted using SMART7 (http://smart.embl-heidelberg.de) and ExPASy software.

### Subcellular localization of Pi54of protein

The deduced amino acid sequence of *Pi54of* was used for the *in silico* analysis using WoLF POSRT software [29]. The mGFP coding sequence was PCR amplified by the exon specific primers and cloned at *Xba*I site of pRT101.1 vector in frame with at N-terminus with *Pi54of* CDS already cloned under the control of CaMV 35S promoter. For localization study, the onion lower epidermis was used for bombardment by a pneumatic particle gun (PDS-1000/He, BIO-Rad, USA) and the cells were observed by a confocal microscope (TCS SP2, Leica, Wetzlar, Germany) following standard protocol [18].

### *In vitro* expression and purification of *Pi54of* protein

The *Pi54of* ORF was PCR amplified and cloned into pET29a vector using gene specific primers with recognition sequences for *Kpn*I and *Sal*I restriction enzymes and transformed into BL21-DE3 bacterial expression systems (Novagen). Expression and protein purification were performed according to instructions provided in the pET System Manual (http://www.novagen.com).

### Characterization of Pi54of protein

The protein concentration was determined by the bicinchoninic acid (BCA) assay [30]. Protein samples and 5X sample loading buffer (Thermo Scientific, USA) were mixed in a ratio of 4∶1 (v/v) in an eppendorff tube, heated to 100°C for 2–5 min and were analysed by 12% SDS-PAGE. The SDS-PAGE resolved Pi54of and Pi54 proteins gel was used for western blot analysis using custom synthesized polyclonal antibodies for Pi54 protein (Sigma, USA) following standard protocol [31]. Further Pi54of was analyzed by MALDI-TOF/TOF with Autoflex, Mass spectrometer MALDI-TOF/TOF instrument (Bruker Daltonics, Billerica, MA, USA) as described by Condina et al. [32]. Peptides from mass spectra of in-gel digested samples were matched against protein databases such as Swissprot, NCBInr, and MSDB using Mascot search engine (Matrix Sciences, London, UK) for peptide mass fingerprinting (PMF).

### Molecular docking of Pi54 and AVR-Pi54 proteins

The 3D models of Pi54 orthologues and AVR-Pi54 was generated by *ab initio* structure prediction method using I-TASSER web based server (zhanglab.ccmb.med.umich.edu/I-TASSER/). The structures were refined by ModRefiner server (http://zhanglab.ccmb.med.umich.edu/ModRefiner/). Energy calculation and minimization was performed using CHARMM force field for 2000 steps [33] (Table S2 in File S1). The potential energy, van der Waals energy and electrostatic energy of *Pi54* orthologues and AVR-Pi54 models was computed using Discovery Studio v 3.5 (http://accelrys.com/products/discovery-studio/). The dependability and quality of these predicted models were ensured by Ramachandran plot analysis (http://www.cgl.ucsf.edu/chimera/download.html). Molecular docking of Pi54 orthologues and AVR-Pi54 was performed using Z-Dock server of Discovery Studio v 3.5 by removing water molecules and adding hydrogen ions based on the CHARMm force field (Table S3 in File S1). The 3D structures and molecules involved in interactions were visualized at 4 Å distance using Accelrys DS 3.5 visualizer software. The relative binding efficiency of AVR-Pi54 with Pi54of, Pi54 and Pi54rh proteins was analyzed.

### Transgenic development and complementation assay

A rice transformation vector (pRiTV) was constructed using pBlueScript SKII (Stratagene, USA) and full-length transcript of *Pi54of* was driven by CaMV35S and terminated at *nos* site. The full cassette was cloned in the *Sma*I site of the multiple cloning sites (MCS). The hygromycin gene (*hptII*) amplified from pCAMBIA 1305.1 was also cloned in the *Sac*II site of MCS. The embryogenic callus derived from mature seeds of rice cultivar IET16310 and TP309 were used for genetic transformation by biolistic approach using helium-driven Particle Delivery System (PDS 1000, BioRad, Washington DC, USA). The calli were regenerated into mature fertile plants following the standard protocol [34]. The putative transgenic rice plants were confirmed by PCR using *Pi54of*, marker gene and 35S-*nos* specific primers and also by Southern blot analysis using DIG labelled probes [31]. Phenotyping of T_1_ generation transgenic IET16310 plants was performed with *M. oryzae* (RML21 and 6-Sikkim) spore suspension, as described earlier. Similarly for phenotyping of T_0_ generation transgenic TP309 plants, detached leaf assay employing young leaves of grown up plants with *M. oryzae* (Sikkim) spore suspension was used [35]. The leaves were placed on the sterilized Whatman No.3 filter paper and 2 µl *M. oryzae* spore suspension (1×10^5^ spores/ml) was placed on leaf surfaces. The whole set up was placed in a humid chamber (95%) at 28°C in dark for initial 24 h and then shifted to 16/8-h light/dark regime. The disease reaction was recorded 7 days after inoculation. Further for resistance spectrum comparison we performed phenotypic analysis of T_1_ generation transgenics of TP309 and IET16310 plants along with independent transgenic TP309 plants containing *Pi54* and *Pi54rh* genes with spray inoculation of *M. oryzae* strain MG-79, which differentiates *Pi54* and *Pi54rh*. The non transgenics lines and highly susceptible rice cv. HR12 were used as controls in all the cases.

### Development of allele specific functional CAPS marker

A functional Cleaved Amplified Polymorphic Site (CAPS) marker was designed using *Hind*III restriction enzyme which digests the ORF of *Pi54of* at 954 bp, but do not digest *Pi54tp* allele of TP309. The PCR fragments amplified using ORF specific primers from transgenic and non-transgenic TP309 plants were analyzed.

## Results

### Expression analysis and cloning of *Pi54of* gene

**Figure 1:**
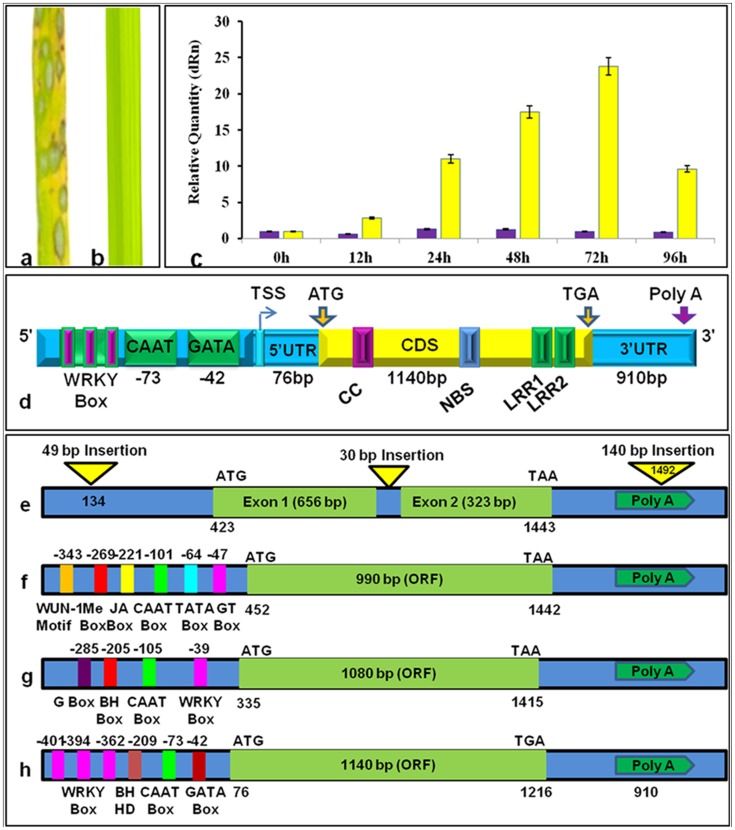
Phenotypic and expression analysis of *Pi54of* gene. Reaction of HR-12 (a) and *O. officinalis* (b) to with *M. oryzae*; c: Relative expression; pre-and-post inoculation of *O. officinalis* with *M. oryzae* using qRT-PCR at different intervals; d: Schematic representation of full length *Pi54of* gene; e: Structural analysis of *Pi54* orthologues in f: Nipponbare; g: Tetep (Sharma et al. 2005); h: *O. rhizomatis* (Das et al. 2012) and i: *O. officinalis*.

The phenotyping results showed that the wild rice *O. officinalis* was completely resistant when inoculated with *M. oryzae* isolate Mo ei-11, whereas the susceptible check rice line HR12 showed scale 4–5 susceptible reaction (Figure 1). A relatively higher cycle threshold (CT) value (33.22) was obtained at 0 hpi of the seedlings of *O. officinalis* indicating low abundance of transcript in the pre-inoculated plants. The relative expression revealed the increased upregulation of *Pi54of* from 24 hpi and reaches maximum expression of 23 folds at 72 hpi before decreasing drastically to 9.6 folds at 96 hpi (Figure 1). For the cloning of full length *Pi54of* gene, heterologous primers were used and a ∼1.2 kb region was amplified. Further iPCR amplification could amplify ∼2 kb flaking region. The 5′ RACE PCR results revealed the position of Transcription Start Site (TSS) at 76-bp upstream of the start codon (ATG) of the *Pi54of* transcript (Figure S1). The sequence reads were assembled to get a high quality (>Phred 30) consensus sequence for predicting full length *Pi54of* (Figure 1). The ORF sequence of the gene is available at EMBL Database (Accession no. HE589448).

### Structural and comparative analysis of *Pi54of*

Among the cloned *Pi54* orthologues, *Pi54of* is the largest coding for 379 amino acids, whereas *Pi54* and *Pi54rh* code for 330 and 360 amino acids, respectively. The ORF region of *Pi54of* showed 80.7% and 84.1% identity at nucleotide and 77.3% and 84% identity at amino acid level with *Pi54* and *Pi54rh*, respectively. The *Pi54of* differed from *Pi54* at 45 transition and 28 transversion sites. Whereas, six transition and transversion sites differentiated *Pi54of* and *Pi54rh* orthologues.

**Figure 2:**
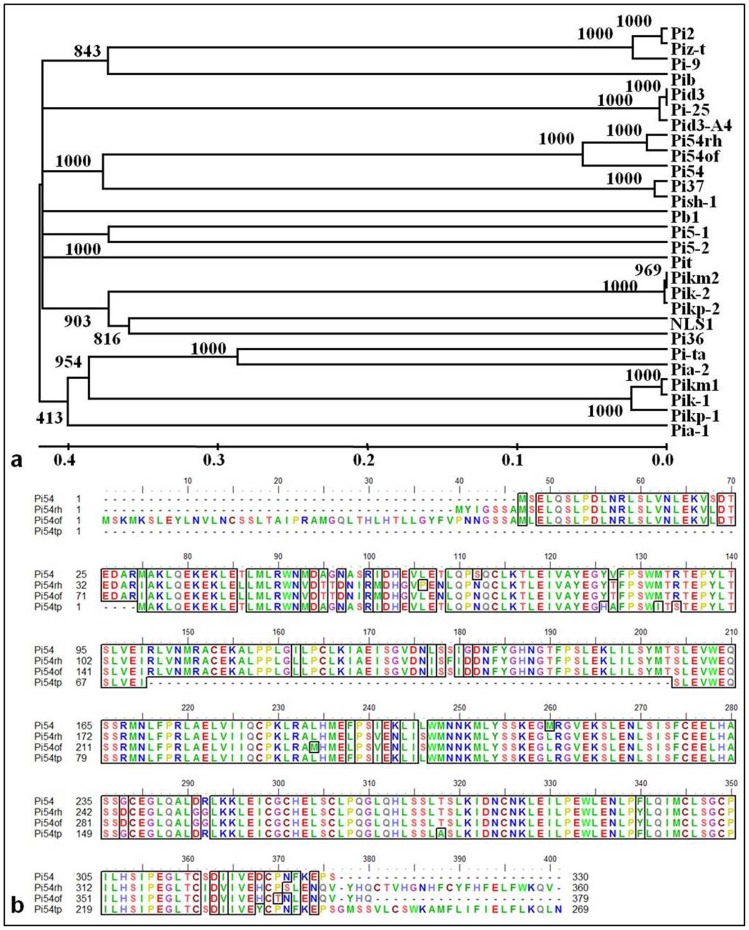
Phylogeny and alignment of amino acid residues from ORF of *Pi54, Pi54of* and *Pi54rh* orthologues. a: Phylogenetic relationship among the cloned rice blast resistance genes. Tree was generated using neighbor-joining method by MEGA.6 software with bootstrap value 1000 replications. The unit of branch length is 0.1 nucleotide substitutions per site, and is indicated by a bar at the bottom left corner of the tree. b: Aligned sequences of Pi54 protein orthologues using BioEdit tool.

Analysis of 1kb promoter region of *Pi54of* indicates presence of GATA (42 bp upstream of TSS), CAAT (73 bp upstream of TSS), BIHD (209 bp upstream of TSS) and three WRKY Box elements (362, 394 and 401 bp upstream of TSS). The structural features of *Pi54* allele from rice cv. Tetep and Nipponbare and the orhologues from *O. officinalis* and *O. rhizomatis* were compared. The susceptible *Pi54* orthologue in cv. Nipponbare has three insertions like 49 bp insertion in the promoter region, a 30 bp insertion in the open reading frame and 140 bp insertions in the Poly-A region. These insertions were not obtained in rest of the alleles. In the *Pi54* gene cloned from rice cv. Tetep, five regulatory elements, WUN-1 motif, MeJA responsive element, CAAT box, TATA box and GT-1 box were predicted in the promoter. In *O. rhizomatis*, four regulatory elements, G-box, BH-box, CAAT box and WRKY box were predicted in the promoter region (Figure 1). The phylogenetic analysis of cloned rice blast resistance gene products revealed divergent clusters. The three *Pi54* orthologues belong to a single sub-cluster and are closely related to two cloned *R*-genes, *Pi37* and *Pish-1* (Figure 2).

### Physico-chemical properties of Pi54 proteins

We predicted that *Pi54of* codes for ∼43 kDa cytoplasmic protein with pI of 5.16. It has functional motifs, such as N-glycosylation site (5 copies), Casein Kinase II phosphorylation site (12 copies), N-myristilation site (4 copies), Protein Kinase C phosphorylation site (3 copies) and Tyrosine Kinase phosphorylation site (1 copy). The multiple sequence alignment of the proteins of *Pi54* orthologues showed a large deletion corresponding to 58 amino acid residues in *Pi54tp* gene (Figure 2). The protein hydrophobicity analysis predicted that the Pi54 proteins of resistant alleles were slightly hydrophilic in nature compared to the Pi54tp protein of susceptible allele. The Pi54 was more hydrophilic followed by Pi54of, Pi54rh and Pi54tp proteins (Figure S2). Present analysis also revealed that Pi54 proteins are relatively thermostable, but overall these all resistance proteins are unstable in nature (Table S4 in File S1).

**Figure 3:**
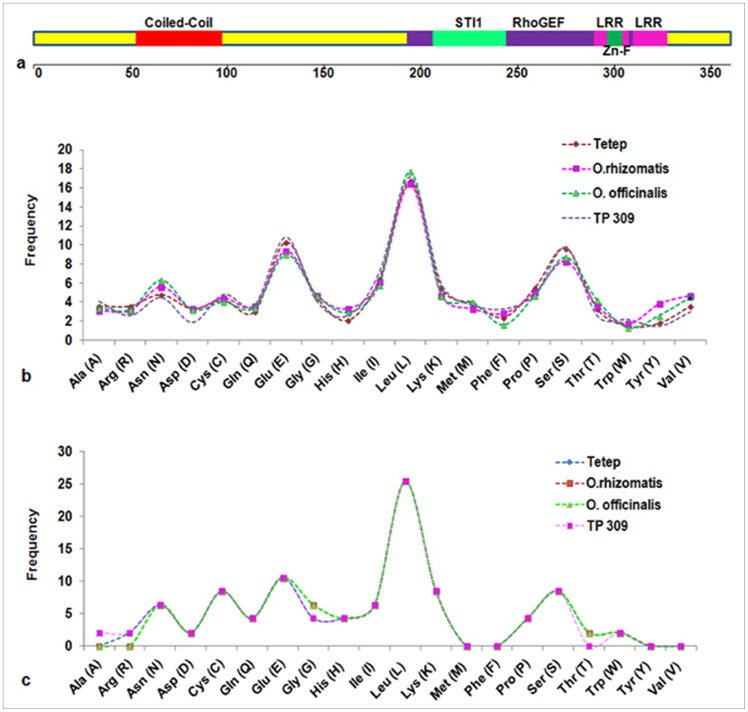
Schematic representation of predicted functional domains in Pi54of protein (a) and the frequency of amino acid residues found in four *Pi54* orthologue proteins; b: orthologue proteins; c: LRR region of all orthologue proteins.

The SMART and ExPASy analysis of Pi54of protein predicted important domains such as, CC, LRR, Zinc finger domain, RhoGEF (Guanine nucleotide exchange factor for Rho/Rac/Cdc42-like GTPases) domain and STI1 domain (Heat shock co-chaperonin-binding motif) involved in the plant defense mechanism (Figure 3). The amino acid composition analysis of the four R- proteins as well as the their LRR domains showed the presence of amino acids Leucine (L), Glutamic acid (E) and Serine (S) in higher frequency (Figure 3). Among the Pi54 proteins, the CC domain was present in the resistant *Pi54* orthologues and absent in *Pi54tp* protein and significance of the prediction was more for Pi54of than Pi54 and Pi54rh.

### *In silico* 3D structure prediction and molecular docking

**Figure 4:**
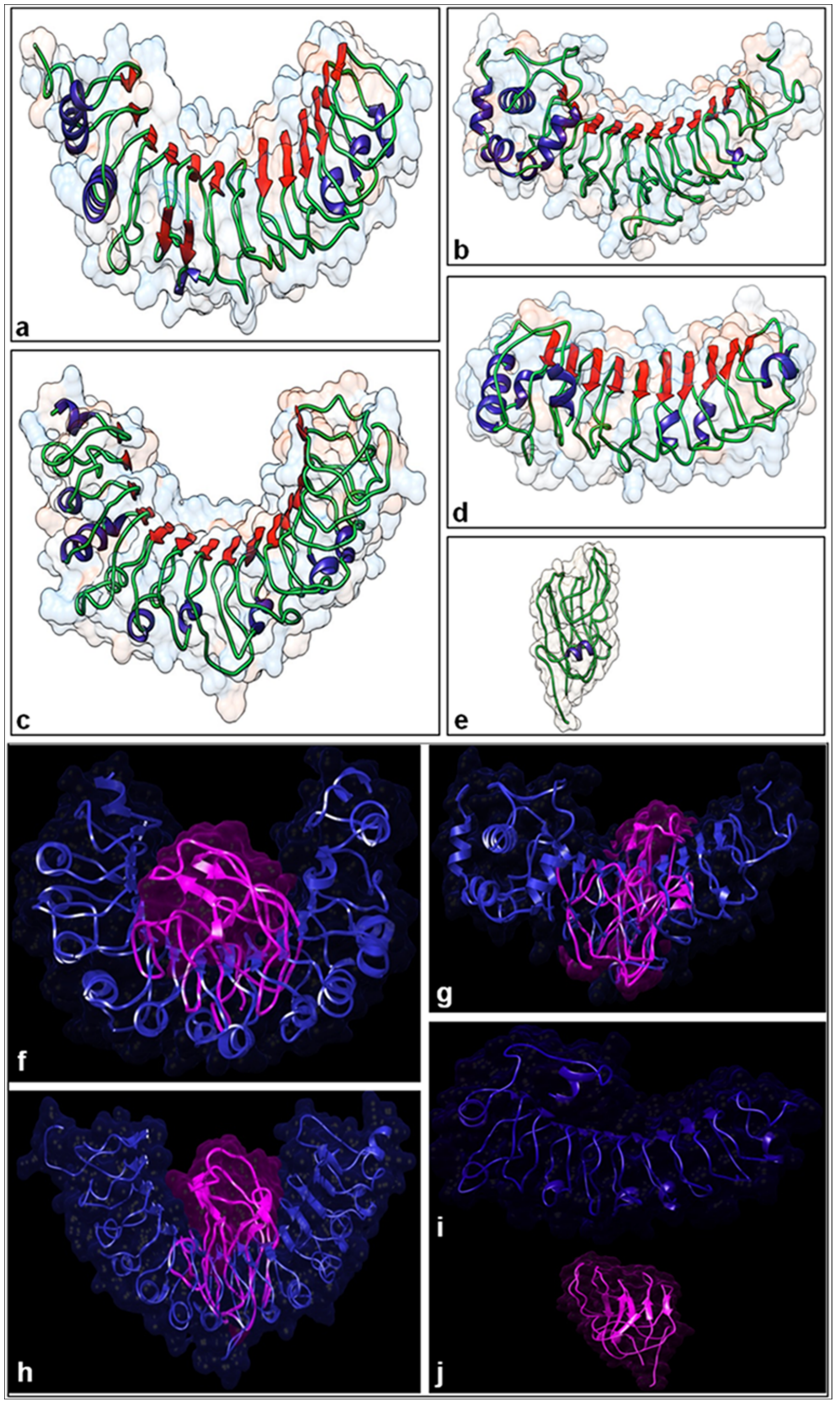
Molecular docking of Pi54 proteins. Predicted tertiary structures of Pi54 orthologue protein Pi54 (a); Pi54rh (b); Pi54of (c); Pi54tp (d) and AVR-Pi54 protein (e) generated by *ab initio* modeling using I-TASSER server. Various secondary structure; Alpha helices (blue), β-sheets (red), Coils (green) have been depicted. Docked AVR-Pi54 with protein Pi54 (f); Pi54rh (g); Pi54of (h) and Pi54tp (i) using Z-DOCK server in Discovery studio 3.5. In the interaction, AVR protein is depicted in pink color and R-protein in blue color.

The 3D structure of Pi54of and other Pi54 orthologues predicted typical horseshoe-shaped conformation (Figure 4). The global free energy of Pi54of protein was found to be much lower than Pi54, Pi54rh and Pi54tp proteins despite being larger in size (Table 1). Ramachandran plot analysis using UCSF-CHIMERA server showed that more than 98% amino acid residues of Pi54 proteins and 89.4% amino acid residues of AVR-Pi54 fell in the most favoured regions and allowed region of the plot (Figure S3). The molecular docking results showed that the binding of AVR-Pi54 with Pi54 was more robust followed by its binding with Pi54of and Pi54rh and no interaction was found with Pi54tp (Figure 4 and Table 1). Analysis of amino acid residues involved in the interactions showed that the Pi54 protein interacts with AVR-Pi54 through its CC, non-LRR and LRR regions. Whereas, Pi54rh interact through its non-LRR and LRR region and Pi54of interacts only through its non-LRR region (Figure 4, Table 2).

**Table 1:** Comparison of various energy values and docking features for each of the best of modelled complex of Pi54 orthologues and AVR-Pi54 using ZDOCKER in DS3.5.

Name	Forcefield	Potential Energy(kcal/mol)	Van der WaalsEnergy (kcal/mol)	Electrostatic Energy(kcal/mol)	TotalEnergy	Binding energy withAVR-PI54 (kcal/mol)
Pi54	CHARMm	−22362.03	−2303.26	−23069.97	−47735.26	−**2265.89**
Pi54rh	CHARMm	−22904.48	−2426.16	−23654.45	−48985.13	−1883.53
Pi54of	CHARMm	−**23888.54**	−**2579.13**	−**24675.68**	−**51143.35**	−1942.56
AVR-Pi54	CHARMm	−7388.96	−778.53	−7751.92	−15919.42	–

Binding energy = energy of complex − [energy (receptor) + energy (ligand)].

**Table 2:** Docking features (hydrogen bond forming residues with bond length <4Å) of modelled Pi54 rthologues and AVR-Pi54.

Gene	Amino acid residues involved in bond formation		
	Coiled Coil	non-LRR	LRR
*Pi54*	T-24, D-26	F-138, Y-139, G-140, H-141, N-142, G-143, F-145,, S-147, L-148,E-149, K-150, L-151, Y-155, M-156, T-157	R-246, K-248 and E-251
*Pi54rh*	–	F-145, Y-146, T-151, F-152, E-156, K-157, L-158, S-161, Y-162,M-163, T-164, S-165	G-253, L-254, L-256, L-257, E-258, I-259,C-260, C-262,S-276, S-277, K-282
*Pi54of*	–	F-191, S-193, L-194, E-195, I-198, L-199, S-200, Y-201 and M-202	–

### *In vitro* expression and characterization of Pi54of protein

**Figure 5:**
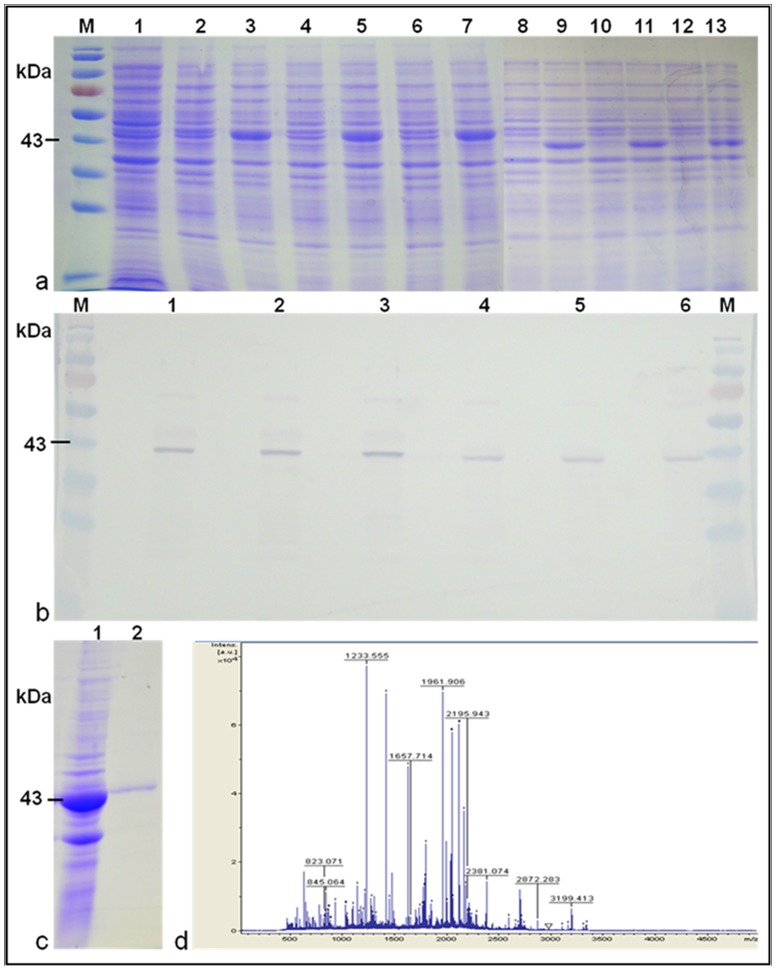
*In vitro* expression and characterization of Pi54of protein. a: SDS-PAGE analysis of IPTG induced BL21 clones. Lane 2–7: *Pi54of* clones; Lane 8–13: *Pi54* clones. b: Western blot of *Pi54of* (1–3) and *Pi54* (4–6) protein using polyclonal Ab developed for Pi54 protein. c: Purified Pi54of protein. d: MS mass spectra MALDI-TOF analyzed Pi54of protein; ID 1233.555 showed match with Pi54 protein.

Protein electrophoresis of IPTG induced and uninduced pET29a clones confirmed the induction of ∼43 kDa and ∼37 kDa protein products in *Pi54of* and *Pi54* clones, respectively (Figure 5). Western blot analysis revealed the hybridization of antibody to ∼43 kDa Pi54of and ∼37 kDa Pi54 proteins (Figure 5). The peptide mass fingerprinting analysis performed using MALDI-TOF mass spectrometry showed match of its spectra with the Pi54 proteins of rice lines resistant to the pathogen (Figure 5).

### Subcellular localization of Pi54of protein

**Figure 6:**
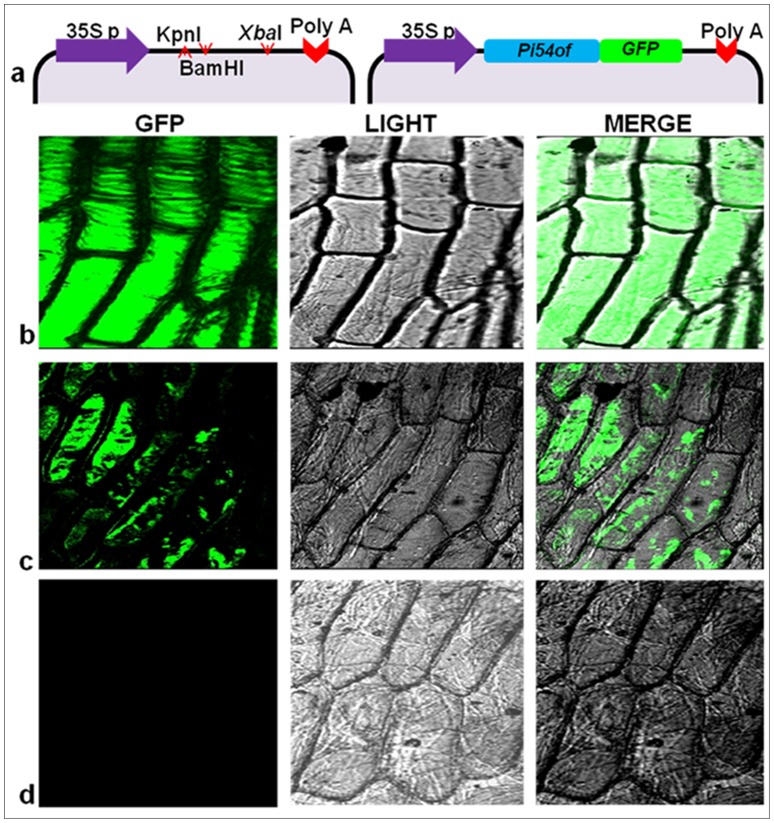
Sub-cellular localization analysis of Pi54of protein. Panel a: Schematic representation of construct used. Panel b: GFP alone under the control of *CaMV 35S* promoter; Panel c: GFP-Pi54of fusion protein under the control of *CaMV 35S*; Panel d- non bombarded onion cells.

For subcellular localization, Pi54of protein was fused with N- terminal of the reporter gene *mGFP* under the control of constitutive CaMV 35S promoter and was expressed transiently in onion epidermal cells (Figure S4). The GFP fluorescence which was observed 24 h post bombardment revealed that Pi54of-mGFP was localized to the cytoplasm, whereas the GFP control was evenly distributed throughout the cell (Figure 6).

### Functional validation of the gene

**Figure 7:**
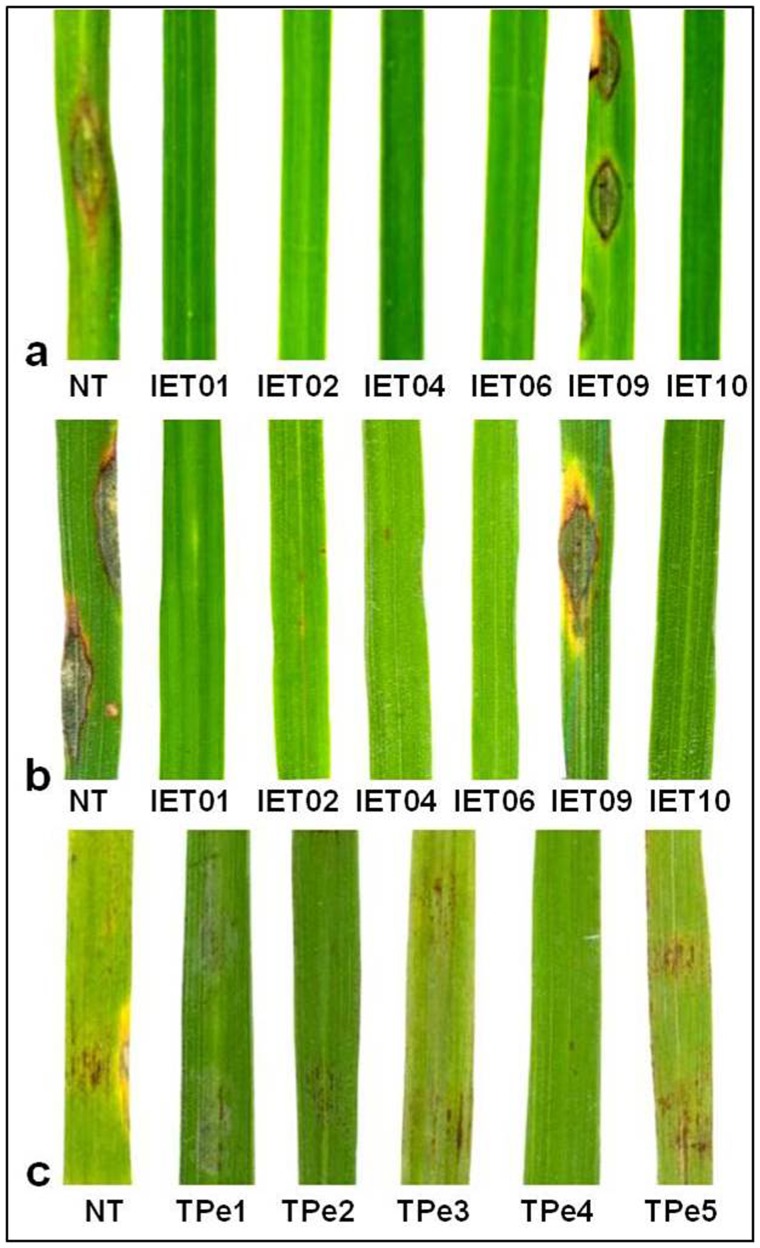
Genetic complementation of transgenic plants. Transgenic IET16310 plants challenged with *M. oryzae* stain RML21 (a) and 6-Sikkim (b). Detached leaves of transgenic TP309 plants challenged with *M. oryzae* (6-Sikkim) (c). NT (non transgenic) plants were used as control in all the experiments.

For functional validation, *Pi54of* gene was cloned in rice transformation vector (pRiTV) and transferred to the rice lines IET16310 and TP309 susceptible to *M. oryzae*. Molecular analysis of the putative transgenics of IET16310 (Figure S5) lines was performed. The PCR amplification showed the presence of 1, 140 bp *Pi54of*-specific and 500 bp *hptII*-specific amplicons in IET16310 transgenic plants (Figure S5). The Southern blot analysis of PCR positive transgenic plants revealed that the plant number IET01, IET02, IET04, IET06 and IET09 had single copy insertion, where as IET10 showed two copies of *Pi54of* integrated in its genome (Figure S5). Similarly, the putative transgenic TP309 plants (Figure S6) were confirmed by PCR to amplify 1,140 bp *Pi54of* specific ORF and ∼1, 851 bp fragment corresponding to *35S-Pi54of-nos* region. Further, the functional allele specific CAPS marker analysis also showed the presence of *Pi54of* gene in the five transgenics of TP309 events and its absence in non-transgenic plants (Figure S6). The phenotyping results of 6 transgenic IET16310 plants showed that, events IET01, IET02, IET04, IET06 and IET10 were resistant to *M. oryzae*, whereas IET09 showed susceptible reaction to the pathogen (Figure 7). Similarly, transgenic TP309 plants were challenged with *M. oryzae* isolate 6-Sikkim and the results showed that all five transgenic TP309 events (TPe1–TPe5) showed hypersensitive reaction (HR), where as non-transgenic control showed typical susceptible blast symptoms (Figure 7). The comparison of resistance response of transgenic TP309 lines containing *Pi54* orthologues revealed that TP-*Pi54* was susceptible and TP-*Pi54rh* and TP-*Pi54of* displayed resistance reaction against *M. oryzae* isolate MG-79. Further all the transgenic of IET series except IET09 harbouring *Pi54of* were resistant to isolate MG-79 (Figure S7).

## Discussion

Over the years rice monocroping has lead to the increased susceptibility of many local rice cultivars to *M. oryzae*. Management of rice blast through host plant resistance is considered as viable and ecofriendly option. Since 1999 when first rice blast resistance gene *Pib* was cloned [36], efforts have been made to successfully clone many resistance genes from different sources. Currently 23 rice blast resistance genes have been cloned and characterized. Owing to high variability in *M. oryzae* pathogen population the average effective life of major rice blast resistance genes is reported to be very short. In view of this, continued identification and characterization of novel genes and its alleles is important for their use in stacking of multiple *R*-genes or transferring broad spectrum disease resistance genes to the cultivated varieties [37]. These cloned *R*-genes have untapped novel alleles in other rice backgrounds, providing useful source for efficient management of the rice blast disease. Some of the alleles of cloned *R*-genes have been reported to provide much stronger and broader resistance against rice blast disease [11], [18]. The rice blast resistance gene *Pi54* provided broad spectrum resistance to *M. oryzae* population prevalent in different parts of India [22]. Allele mining for *Pi54* gene has resulted in the isolation and cloning of few better performing alleles and orthologues from various rice lines and wild rice species, respectively [18].

In the present investigation, an orthologue of *Pi54* designated as *Pi54of* (of-*officinalis*) was cloned from the wild rice species *O. officinalis*, characterized and functionally validated. The expression of *Pi54of* in the broad spectrum blast-resistant wild species of rice, *O.officinalis* pre-and-post challenge with *M. oryzae* found to be at the basal level before infection, but it showed highly induced expression at 72 hpi. This is similar to the expression pattern of *Pi54* gene, which is reported to be induced only upon pathogen challenge [21] and the expression of *Pi54rh* gene that was reported to be highly upregulated at 72 hpi [18]. The other blast resistance gene *Pib*, showed induction upon altered environmental conditions besides pathogen inoculation [36]. Similar upregulation was observed for rice bacterial blight resistance gene *Xa1* which showed induction upon pathogen challenge and wounding [38]. The late upregulation of the *Pi54of* gene might be attributed to its role in limiting the spread of pathogen to the cells adjacent to the site of infection and also secondary spread of fungal biomass at later stages [39] or by fortifying the membrane and plasmodesmata [40]. The rice blast resistance gene *Pi54* has also been reported to trigger the upregulation of five defence response genes (callose, chitinases, laccase, PAL and peroxidise) and defence related transcription factors (NAC6, Dof Zinc Finger, MAD Box, bZIP and WRKY) which results in blast resistance phenotype in rice [41].

The genetic variability of *Pi54of* at both the nucleotide and amino acid levels indicated its divergent nature from the *Pi54* orthologue. Among these orthologues, *Pi54of* is the largest and its single exon coding for 379 amino acids. The analysis of promoter region of the *Pi54of, Pi54* and *Pi54rh* shows the presence of CAAT Box in *Pi54of* and *Pi54rh* orthologues and TATA Box in *Pi54* gene. Besides, *Pi54of* has three WRKY71OS boxes, whereas *Pi54* and *Pi54rh* have one WRKY71OS box each. This cis-acting region is found in the Pathogenesis-Related Class10 (PR-10) genes [42]. The WRKY boxes are also identified in the promoter regions of the defence response genes like *NPR1* gene of *Arabidopsis*, Class I chitinase gene, *CHN50* in tobacco and parsely *PR-1* gene [18]. In case of rice, it has been reported that WRKY transcription factor acts as an activator of defence related genes after inoculation with *M. oryzae* [43] and rice OsWRKY76 expresses within 48 h after inoculation with *M. oryzae* [44]. BELL homeodomain (BH) box predicted in the *Pi54of* is specialized nucleotide sequences reported to be involved in the upregulation of defence response genes and is associated with the blast resistance response in rice [45]. The polyA tail predicted for *Pi54of* is longest of the three *Pi54* genes at 910 bp downstream to stop codon, which is important for nuclear export, stability and translation of mRNA.

The amino acid sequence analysis of Pi54of protein showed the presence of a CC domain, a Zinc finger domain, two LRR domains, apart from N-glycosylation, myristilation and phosphorylation sites. However no NBS domain was predicted in *Pi54of* protein. The previous studies involving *Pi54* and *Pi54rh* however predict a rudimentary NBS domain in both the orthologues using homology based approach and ExPASy tools, respectively [21], [18]. Presence of a phosphorylation site suggests that the protein of this allele has important role in signal transduction and transmittance to the downstream genes. The CC domains predominant in *R*-genes from monocotyledons are the mediators in the recognition and activation of *R*-genes [46]. The LRR domain is the major determinant of recognition specificity of the pathogen avirulence factors. The leucine rich repeats are proven to be the mediators of interaction with Avr-proteins, in a typical protein-protein interaction leading to host resistance [47]. The Zinc finger domain found is unique among the other rice blast resistance gene and might play an important role in broad-spectrum resistance nature of *Pi54of* gene [48].

The evolutionary analysis performed in this study showed the structural relatedness and phylogenetic relationships among cloned *R*-genes. In the present study, a phylogenetic tree was constructed using cloned rice blast *R*-genes including *Pi54of*. The tree depicts the divergent origin of *R*-genes suggesting their evolutionary advantage for recognizing, binding and defending against a broad array of *M. oryzae* isolates [49]. The definitive clustering in the tree showed the common ancestral origin of these *R*- genes which have evolved by duplications and subsequent diversification leading to wide variation in their structure and specificity [50]. The clustering of *Pi54of* with *Pi54* and *Pi54rh* proves the orhologues nature of these three genes and clustering of *Pi54of* with other NBS-LRR type of blast resistance genes in sub-cluster III reveals that *Pi54of* is closely related to the NBS-LRR family of *R*-genes.

*In vitro* expression and characterization of the protein product of target gene is the first step in unravelling the molecular mechanism played by the corresponding gene in the living system. In rice, disease resistance related genes like *Pita-LRD, OsBIHD1, OsCDR1, OsPR-10*, *OsBBI1*, *Pb1* and *RGA5* [51], [45], [52], [53], [54], [16]; [19] have been expressed *in vitro* and characterized. In the present study, *in vitro* expressed Pi54of protein was used for western blot hybridization and MALDI-TOF-MAS spectrometry. The above results confirmed that *Pi54of* is an orthologue of cloned and characterized *Pi54* gene.

Subcellular localization of R-proteins is an important factor which determines the site of interaction of R-proteins with the pathogen effector proteins in a typical gene-for-gene manner. Resistance proteins are localized to the cytoplasm, nucleus or both. Cytoplasmic localization of R-proteins enhances signaling mechanism related to HR response. Whereas, nuclear localization was the part of complete resistance reaction system [55]. In many cases, the R proteins are found to accumulate in the nucleus in response to the pathogen infection [56]. It has been reported that the nuclear localization is required for resistance response of R-proteins like, barley mildew A (*MLA*) 10, tobacco N, *Arabidopsis* RPS4 and RPS6 and rice blast resistance Pb1 [57], [19]. The rice blast resistance protein Pb1 is found in both cytoplasm and nucleus, but nuclear localization of Pb1 was proved to be essential for blast resistance. In the present investigation, Pi54of protein was localized to the cytoplasm which is a subcellular localization site for most of the reported R-proteins. The protein product of the rice blast resistance gene *Pi37* which forms cluster with *Pi54of* was also found to be localized to the cytoplasmic region [58], whereas *Pi-d2* and *Pi54rh* showed their localization to the plasma membrane [18].

In this study, *in silico* comparative analysis of resistant Pi54, Pi54rh and Pi54of proteins along with susceptible protein Pi54tp was performed. Multiple sequence alignment of the four Pi54 proteins revealed the deletion of a large middle fragment between CC and LRR domain in *Pi54tp* which might be contributing to the susceptible nature of the rice line TP309 to *M. oryzae*. The amino acid composition analysis of the four R-proteins as well as their LRR domains revealed a high frequency of amino acids L, E and S. Leucine belongs to the aliphatic amino acids which would contribute to the hydrophobic interaction and to maintain the conformational stability in the interiors of proteins [59]. Hence, presence of higher L in Pi54of protein helps in maintaining 3D conformation and its stability. The charged amino acid, like E contribute to the electrostatic interaction, which is an important force for maintaining structural conformation and its stability in the outer part of the proteins [60]. The presence of this residue in the Pi54of protein makes it more stable in various stress conditions. The conformational stability of the LRR domain is important for interaction of R-protein with Avr proteins of the pathogen *M. oryzae* [5]. The aliphatic index which is >104 for these Pi54 orthologues further support the hypothesis that they are thermostable in nature. The other amino acid S, present in these proteins is the best known residue interacting with the water molecules surrounding the protein due to its hydrophilic nature [61]. Presence of higher S residues may help proteins to interact with the surface water molecules through hydrogen bond formation and helps for the stability of proteins at interacting surfaces [62]. Higher proportion of hydrophilic amino acids may facilitate the interaction of a protein with the water molecules and can form hydrogen bonds [63]. The physico-chemical analysis in the present study showed that resistant Pi54 proteins were comparatively more hydrophilic in nature than the susceptible Pi54tp protein. Hence, the interaction of the resistant Pi54 proteins with water molecules might be much better than the susceptible Pi54tp protein.

The plant NBS-LRR proteins form typical horseshoe like configuration *in vivo*. The crystal structures of more than 20 characterized LRR proteins have revealed that LRR domains typically contain a series of β- sheets, which form the inner surface of horseshoe like configuration [64]. *In silico* 3D conformation prediction Pi54, Pi54rh, Pi54of and Pi54tp proteins display typical horseshoe like structures. The parallel β-sheets formed the concave surface of the horseshoe structure, whereas the α-helices line the convex plain of these proteins. The authenticity of 3D structures was confirmed by Ramachandran plot analysis. It is known that, a good homology model should have more than 90% of its amino acid residues in the favourable region [65]. The Pi54 proteins of all the orthologues examined in this study have >98% of residues in the most allowed and allowed regions, while AVR-Pi54 proteins has 89.4% residues in the most allowed and allowed regions. Ordered regions are often termed globular and typically contain regular secondary structures packed into a compact globule [66] whereas the disordered regions are linear stretches of amino acids. Our GlobPlot-2 analysis showed the presence of three disorder regions at the N-terminal of Pi54rh and may be contributing to distorted horseshoe conformation of Pi54rh protein at the N-terminal end, which may be contributing to its higher global free energy and also comparatively more binding energy with AVR-PI54 protein.

AVR proteins are perceived by R-proteins through direct or indirect interactions. Direct protein-protein interaction has been reported for nine R-proteins with cognate effector molecules [16]. In case of rice-*Magnaporthe* pathosystem, experimental findings showed that recognition of AVR-Pita, AVR-Pia, AVR-Pik/km/kp, and AVR-CO39 occurs inside the host cells by their corresponding cytoplasmic R-proteins [16]. The molecular mechanism of interaction is reported for *AVR-Pita*, *AVR-Pik* and *AVR-CO39*. AVR-Pita interacts with C-terminal LRD domain of Pi-ta whereas, AVR-Pik interacts through the N-terminal domain including the CC domain of Pik-1, and sequences upstream of the NBS domain. The AVR-CO39 protein interacts with a novel non-LRR domain of RGA5A. Hence, the above examples demonstrate the cases of direct recognition wherein it seems to associate different R-protein domains with different resistance mechanisms. The molecular docking results of this study also revealed that the Pi54of interacted with AVR-Pi54 only through its novel non-LRR region corresponding to STI1 and GEF domains, whereas Pi54 and Pi54rh interacted through CC-non-LRR-LRR regions and non-LRR-LRR regions, respectively. The susceptible Pi54tp protein did not show interaction with AVR-Pi54. This differential preference of AVR protein for binding domains among the Pi54 proteins might be attributed to different resistance mechanisms against *M. oryzae*. The docking results of present study correlate well with the phenotypic reaction of all the rice genotypes containing Pi54 proteins with *M. oryzae* [21], [18]. However, proteins coded by late blight resistance *RB* gene of potato and blast resistance genes *Pik* and RGA5A from rice showed that LRR regions need not be the site of interaction with AVR proteins [16].

In the present study two domains involved in the plant defensome complex were predicted. The one being STI1was predicted in Pi54of and the other one was Guanine Nucleotide Exchange Factor (GEF) found in both Pi54of and Pi54rh proteins. STI1 domain is a cochaperone STI1 binding domain. STI1 is a homologue of human cognate protein 70 (hsc70)/heat shock protein 90 (hsp90)-organising protein (Hop) and is a cochaperone for HSP70 and HSP90 [67]. There are two Sti1 homologues in the rice genome. The RhoGEF is also called Dbl-homologous (DH) domain. The presence of STI1 and RhoGEF domains together in *Pi54of* is of considerable significance as these two domains are part of the plant defensome complex [68]. The OsRac1 which is bound to Sti1 is initially in a GDP bound inactive state. The activation of OsRac1 needs a GEF molecule, which binds to RhoGEF domain. The pathogen derived PAMPs such as chitin, induce the activation of GEF and the functional GEF activates OsRac1. Further the receptor of PAMPs, a chitin receptor OsCERK1 transfer the signal to downstream target through Hop/Sti1a and Hsp90 complex which interact with OsCERK1 through its transmembrane domain [68]. The active OsRac1 then recruits RACK1A and RACK1A further interacts with N-terminus of RAR1 and SGT1 and also regulates ROS production by interacting directly with the N-terminus of Rboh, an NADPH oxidase [69]. Furthermore, the activation of rice MAPK6 requires OsRac1 for PAMP-induced regulation and it forms a complex with MAPK6 in rice cell extracts [70]. Considering the importance of stability and spatial arrangements of the components of plant defensome complex, Sti1 found in *Pi54of* might be stabilizing the interaction of kinases and HSP90 in MAPK cascade. The rice blast resistance gene *Pia* pathway was found to be affected by loss of Sti1 [67].

The genetic complementation study, which was performed to understand the functional role of *Pi54of* gene showed that the two susceptible rice lines IET16310 (indica) and TP309 (japonica) transformed with *Pi54of* gene could displays high level of resistance to highly virulent strains of *M. oryzae*. This analysis showed that *Pi54of* is a functional orthologue. The resistance spectrum comparison of independent transgenic TP309 plants harbouring *Pi54* and its two orthologues revealed that three orthologues have different resistance response to *M. oryzae* isolate MG-79 which might be due to their differential interaction with *AVR-Pi54* [18]. The functional Cleaved Amplified Polymorphic Site (CAPS) marker developed in this study, that differentiates the transgenic TP309 plants from non-transgenic lines as well as transgene integrated homozygous plants from heterozygous plants can be used for molecular breeding to analyze *Pi54of* integration and to differentiate homozygous and heterozygous TP309 plants. These findings showed that the cloned *Pi54of* gene provides resistance against three highly virulent strains of *M. oryzae* to two rice lines belonging to both indica and japonica genetic background.

**Figure 8:**
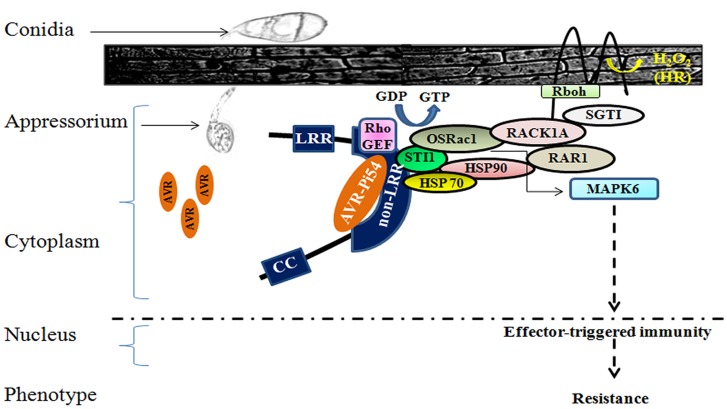
Possible molecular mechanism of *Pi54of* gene. Avr-Pi54 binds to Pi54of protein (blue) at the non-LRR region upstream to the LRR domain. Pi54of may perceive pathogen signals through STI1 which acts as an anchor for the so called defensome complex involving Os Rac1 (Rac/Rop GTPase), RACK1A (Receptor of Activated C Kinase), RAR (Required for Mla12 Resistance), SGT1 (Suppressor of the G2 allele of skp1), MAPK6 (a rice Mitogen-Activated Protein Kinase), Rboh (NADPH oxidases).

The present study provides a new blast resistance gene *Pi54of*, cloned and functionally characterized from wild rice species *O. officinalis*. This gene product is targeted to cytoplasm and is the longest among all the functionally validated *Pi54* genes. The resistance mechanism of the *Pi54of* might be different from other cloned *Pi54* orthologues due to its differential binding pattern with *AVR-Pi54*. It was also found that Pi54of protein interact with the AVR protein through its novel non-LRR region and might be involved in rice innate immunity by forming a so called defensome complex (Figure 8). This study is the first attempt in unravelling the actual molecular mechanism of *Pi54of* during its expression against virulent isolate of *M. oryzae* in two rice lines belonging to indica and japonica genetic background.

## Supporting Information

Figure S1 Cloning of full length Pi54of orthologue from *O. officinalis*.
(TIF)

Figure S2 Composition of hydrophobic and hydrophilic amino acid residues of proteins of Pi54 orthologues.
(TIF)

Figure S3 Ramachandran plot validation of 3D structures of Pi54 orthologue proteins and AVR-Pi54 protein.
(TIF)

Figure S4 Construction of plant expression vector for sub-cellular localization of Pi54-of protein.
(TIF)

Figure S5 Genetic transformation of indica rice line IET16310 and molecular confirmation.
(TIF)

Figure S6 Genetic transformation of japonica rice line Taipei 309 and molecular confirmation.
(TIF)

Figure S7 Phenotypic reaction of *Pi54* orthologues in the TP309 genetic background challenged with *M. oryzae* isolate MG-79.
(TIF)

File S1 Supporting Tables.
(DOCX)
