# Supplementary figures and images for: The Blast Resistance Gene Pi54of Cloned from Oryza officinalis Interacts with Avr-Pi54 through Its Novel Non-LRR Domains
Source: PLoS One. 2014 Aug 11;9(8):e104840. doi: 10.1371/journal.pone.0104840 (PMC4128725; doi:10.1371/journal.pone.0104840)

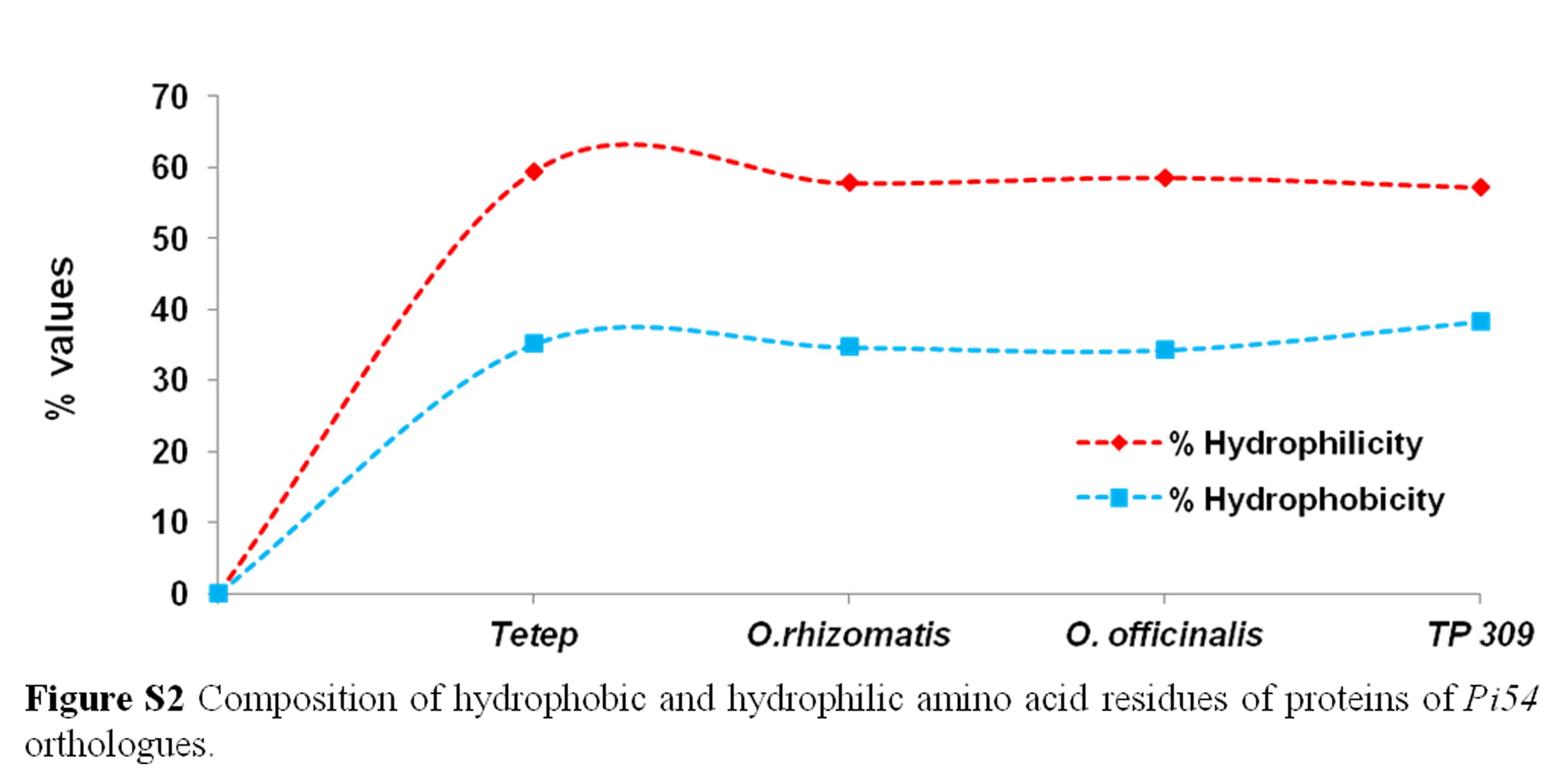

Supplement: Figure S2 — Composition of hydrophobic and hydrophilic amino acid residues of proteins of Pi54 orthologues. (TIF) [file pone.0104840.s002.tif]

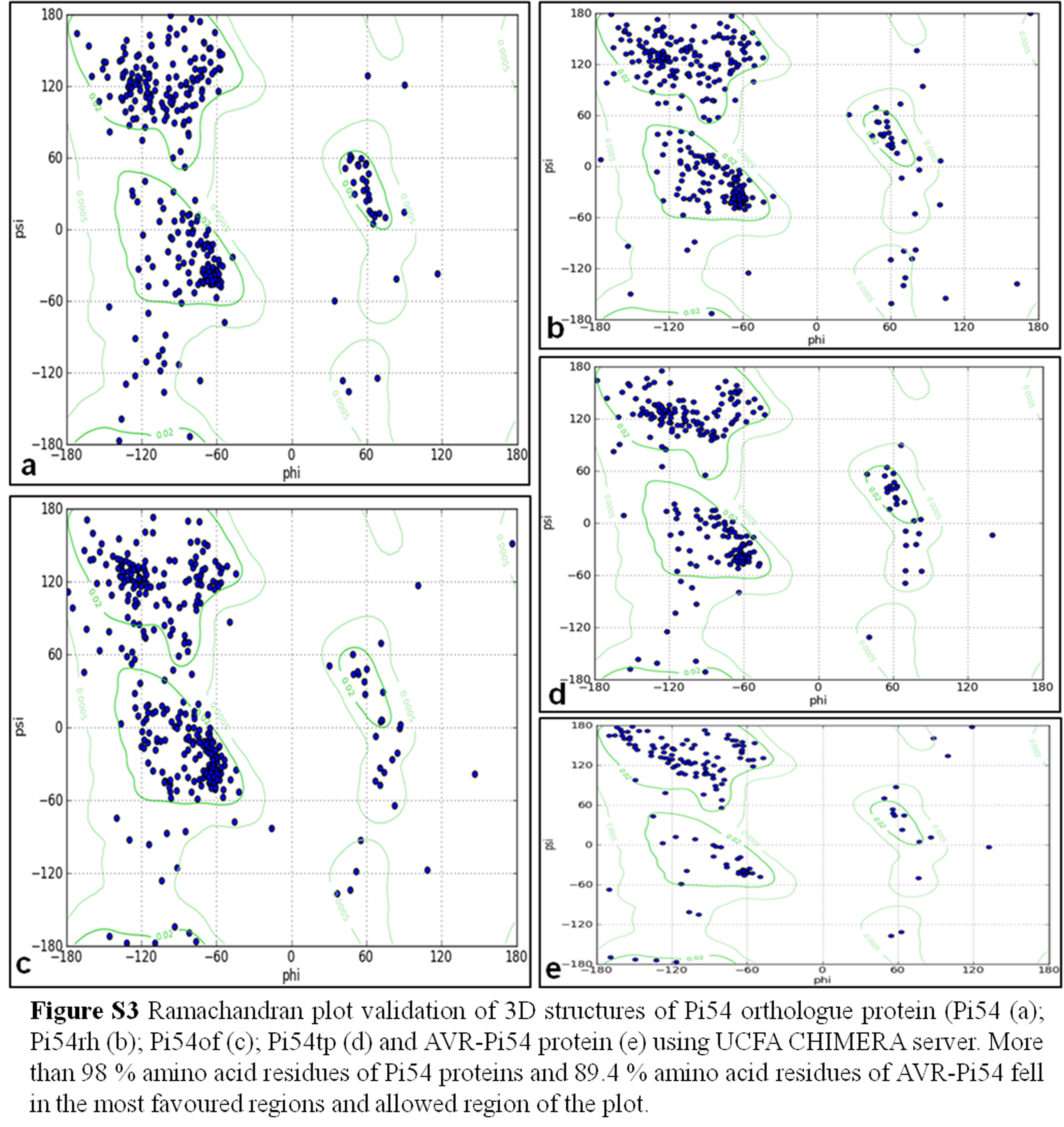

Supplement: Figure S3 — Ramachandran plot validation of 3D structures of Pi54 orthologue proteins and AVR-Pi54 protein. (TIF) [file pone.0104840.s003.tif]

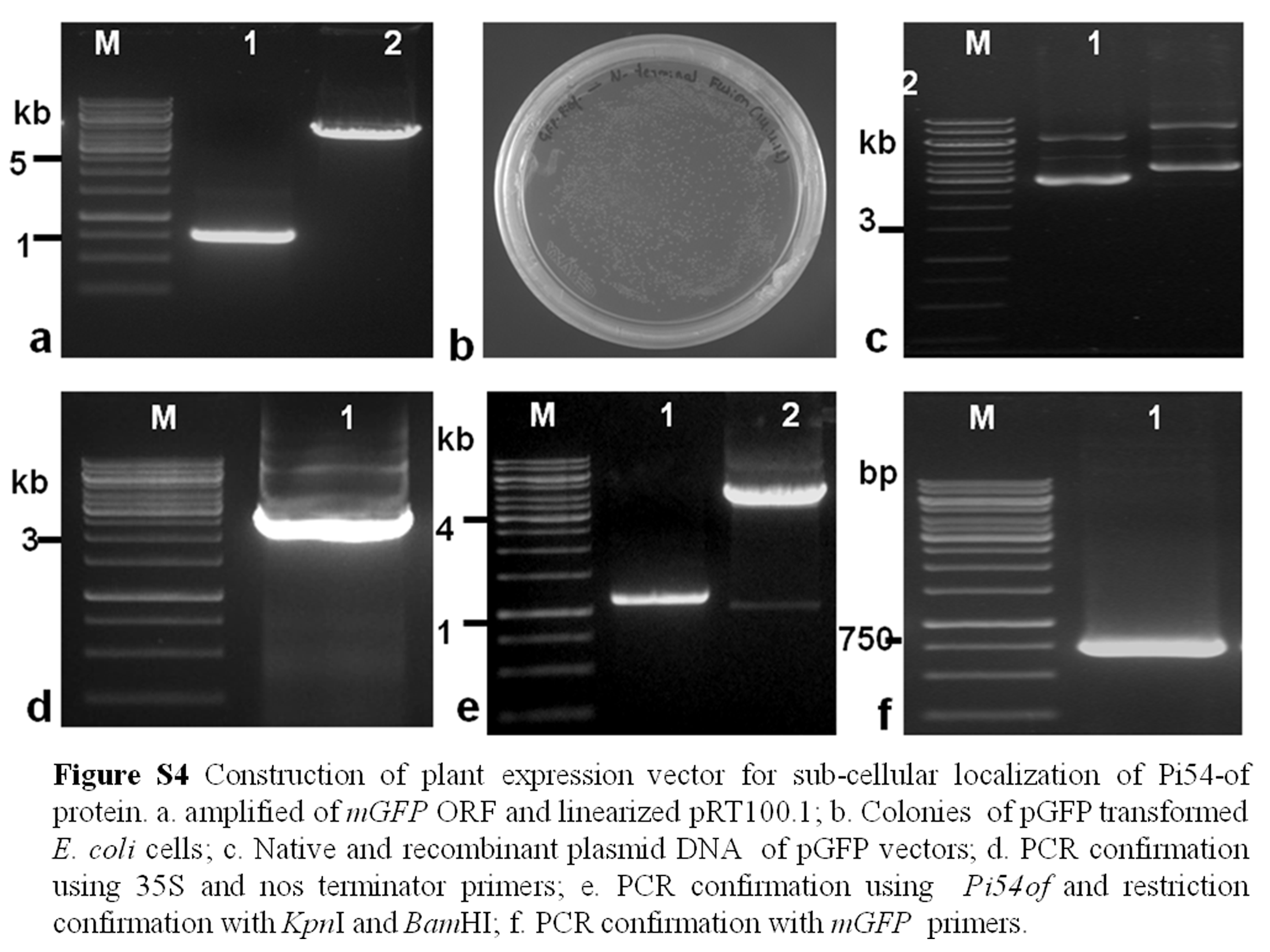

Supplement: Figure S4 — Construction of plant expression vector for sub-cellular localization of Pi54-of protein. (TIF) [file pone.0104840.s004.tif]

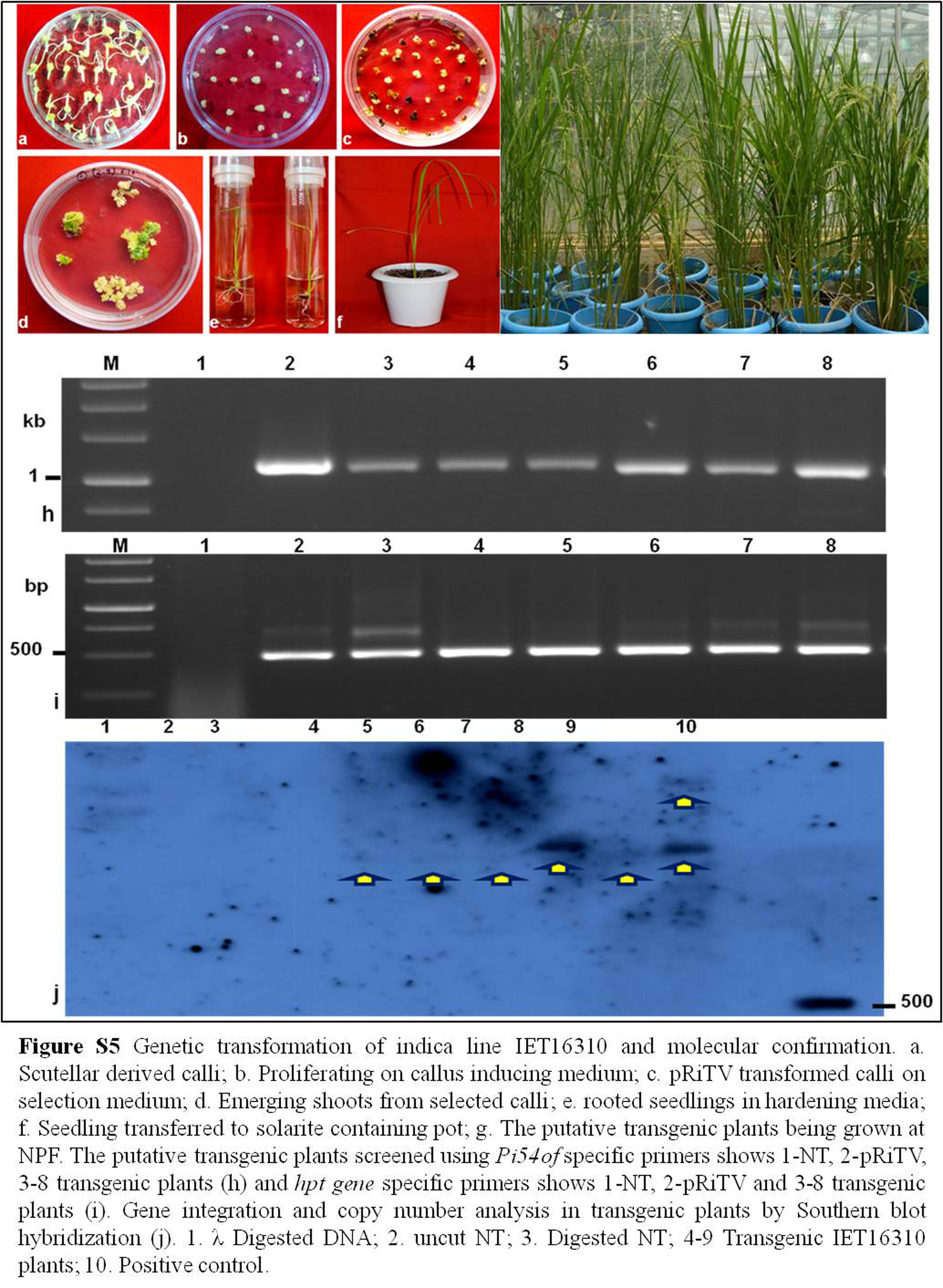

Supplement: Figure S5 — Genetic transformation of indica rice line IET16310 and molecular confirmation. (TIF) [file pone.0104840.s005.tif]

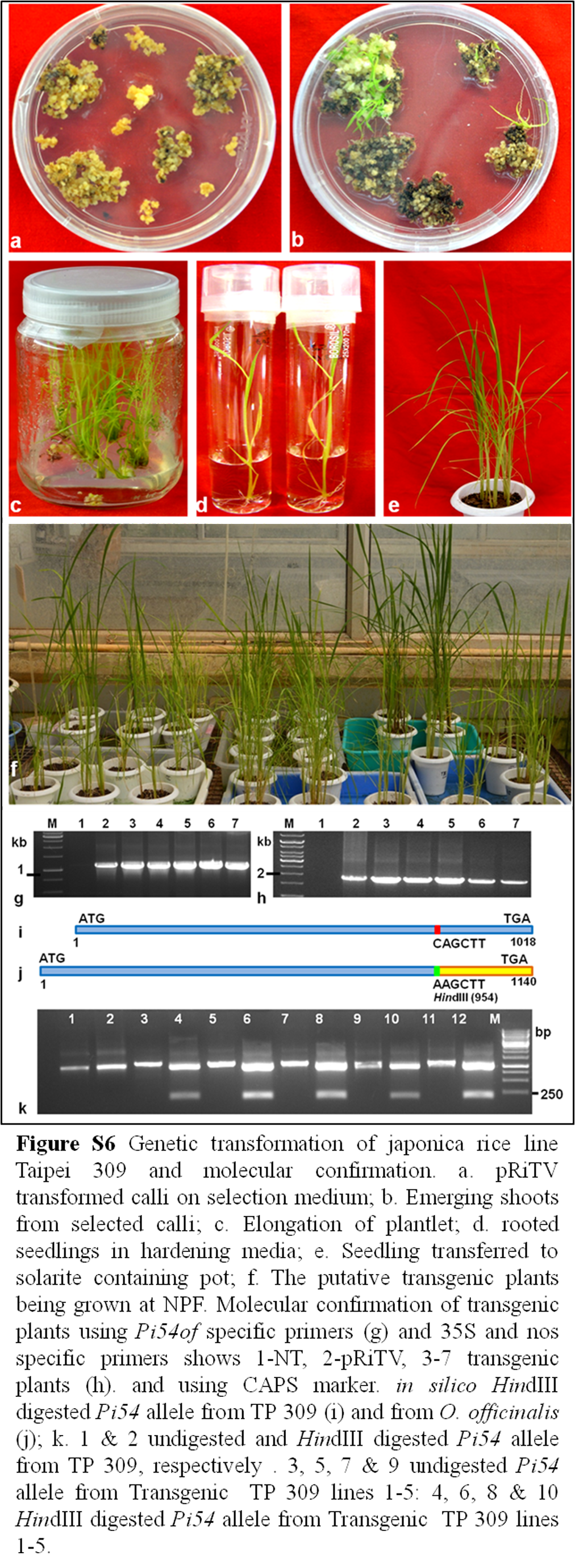

Supplement: Figure S6 — Genetic transformation of japonica rice line Taipei 309 and molecular confirmation. (TIF) [file pone.0104840.s006.tif]

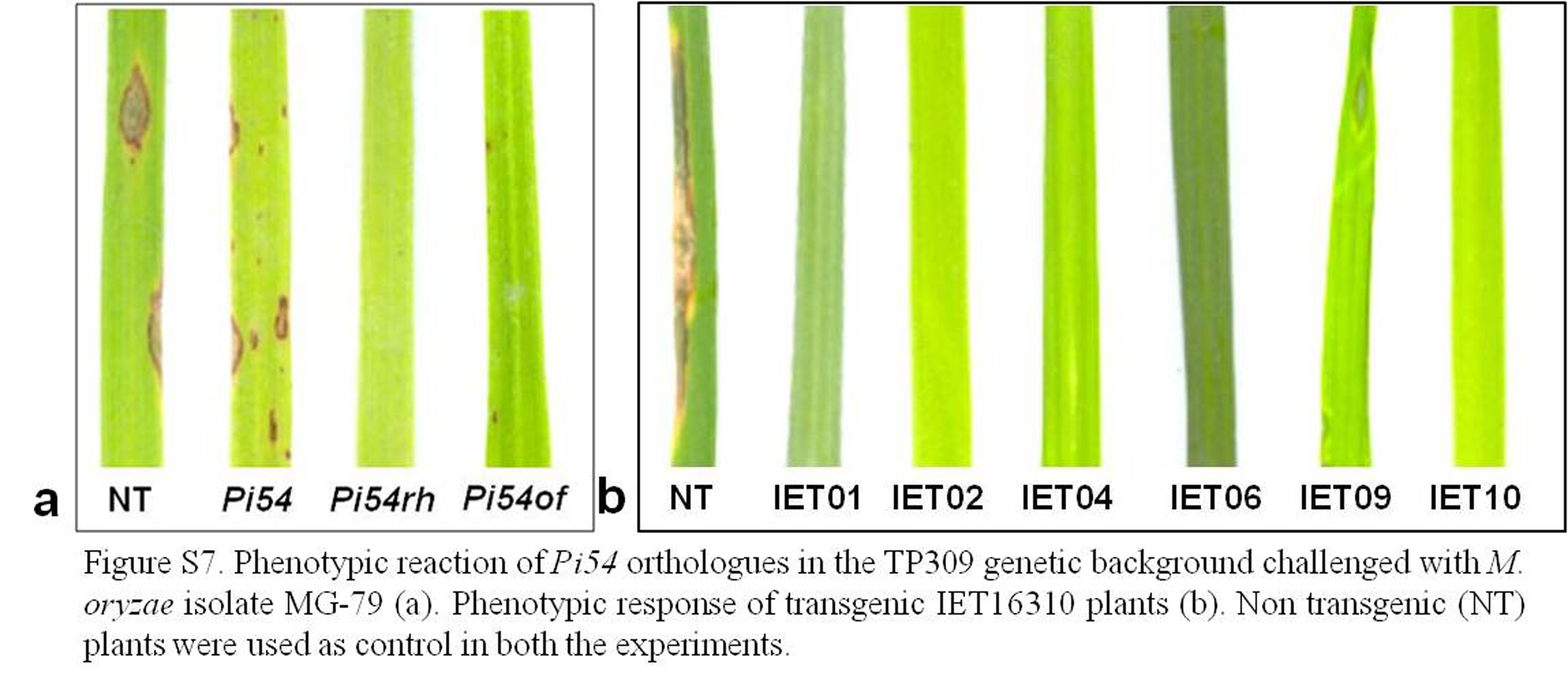

Supplement: Figure S7 — Phenotypic reaction of Pi54 orthologues in the TP309 genetic background challenged with M. oryzae isolate MG-79. (TIF) [file pone.0104840.s007.tif]
