# Supplementary material for: The Blast Resistance Gene Pi54of Cloned from Oryza officinalis Interacts with Avr-Pi54 through Its Novel Non-LRR Domains
Source: PLoS One. 2014 Aug 11;9(8):e104840. doi: 10.1371/journal.pone.0104840 (PMC4128725; doi:10.1371/journal.pone.0104840)
Supplement: File S1 — Supporting Tables. (DOCX) [file pone.0104840.s008.docx]

Table S1. List of primers used in the present study (5′-3′)

| **No.** | | | | **Name** | **Sequence** | | | **Tm (^o^C)** |
| --- | --- | --- | --- | --- | --- | --- | --- | --- |
| **Oligos for allele amplification** | | | | | | | | |
| 1 | ORF-F2 | | | | | | GTCAGGTACCATGAGTAAAATGAAGAGCCTTG | 61.8 |
| 2 | ORF-R2 | | | | | | GTACGGATCCTCACTGATGATATACCTGGTTTTC | 63.2 |
| **Oligos for qRT-PCR** | | | | | | | | |
| 3 | | | | Real F2 | | | TGCAAGAATGGCAAAACTTC | 54.3 |
| 4 | | | | Real R2 | | | ATGGCTCGGTTCTTGTCATC | 58.4 |
|  | | | | EFα1-F | | | TTTCACTCTTGGTGTGAAGCAGAT | 62.0 |
| 6 | | | | EFα1-R | | | GACTTCCTTCACGATTTCATCGTAA | 62.5 |
| **Oligos for 5′RACE** | | | | | | | | |
| 7 | | | | GSP1 | | | ATGGCTCGGTTCTTGTCATC | 58.0 |
| 8 | | | | GSP2 | | | GGAAATGTATACCCTTCATATG | 58.0 |
| 9 | | | | GSP(3) | | | GTAGCCCAAGTGGAGGTAATGC | 58.0 |
| **Oligos for construction of plant transformation vector** | | | | | | | | |
| 10 | | | | HygF2 | | | ATACCGCGGACAGGCAGCAACGCTCTG | 8.4 |
| 11 | | | | HygR2 | | | ATACCGCGGCACGACACTCTCGTCTAC | 6.3 |
| 12 | | | | SMAI F | | | ACCCGGGAAGCTTGCATGCCTG | 8.0 |
| 13 | | | | SMAI R | | | ACCCGGGAAGCTTGCATGCCTG | 8.0 |
| **Oligos for screening of putative transgenics** | | | | | | | | |
| 14 | | | | ORF-F2 | | | GTCAGGTACCATGAGTAAAATGAAGAGCCTTG | 61.8 |
| 1 | | | | ORF-R2 | | | GTACGGATCCTCACTGATGATATACCTGGTTTTC | 63.2 |
| 16 | | | | Hyg-F | | | GGCGAGTACTTCTACACAGC | 58.4 |
| 17 | | | | Hyg-R | | | ATGTCCTGCGGGTAAATAGC | 56.3 |
| 18 | | | | SmaI F | | | ACCCGGGAAGCTTGCATGCCTG | 58.0 |
| 19 | | | | SmaI R | | | ACCCGGGAAGCTTGCATGCCTG | 58.0 |
| **Oligos for inverse PCR** | | | | | | | | |
| 20 | | | | IL1 | | | G GTT GGA GGT TTT CAG GTA CTC CAT GAT | 60.0 |
| 21 | | | | IR1 | | | T TTC TGT GAA GAG TTG CAT GCT TCT TCA | 60.0 |
| **Oligos for *in vitro* expression** | | | | | | | |  |
| 22 | | | Pi4ofKpnI-F | | | GTCAGGTACCATGAGTAAAATGAAGAGCCTTG | | 58.0 |
| 23 | | | Pi4of SalI-R | | | GTCAGTCGACCTGATGATATACCTGGTTTTC | | 58.0 |
| **Oligos for subcellular localization studies** | | | | | | | |  |
| 24 | | GFP-F | | | | TATCTAGAATGGTAGATCTGACTAGTAAAGGAG | | 59.4 |
| 25 | | GFP-R | | | | ATTCTAGATCACACGTGGTGGTGGTGG | | 61.3 |

Table S2. The values of parameters used for energy minimization of Pi4 proteins in CHARMM force field

| **Parameters** | **Values** |
| --- | --- |
| Minimization RMS Gradient | 0.1 |
| Minimization Energy Change | 0.0 |
| Implicit Solvent Model | None |
| Dielectric Constant | 1 |
| Implicit Solvent Dielectric Constant | 80 |
| Minimum Hydrogen Radius | 1.0 |
| Use Non-polar Surface Area | True |
| Non-polar Surface Constant | 0.92 |
| Non-polar Surface Coefficient | 0.0042 |
| Salt Concentration | 0.0 |
| Input Atomic Radii | Van der Waals radii |
| Non-bond List Radius | 14.0 |
| Non-bond Higher Cut-off Distance | 12.0 |
| Non-bond Lower Cut-off Distance | 10.0 |
| Electrostatics | Spherical Cut-off |
| Kappa | 0.34 |
| Order | 4 |
| Apply SHAKE Constraint | True |

Table S3. The parameters and their values used for performing docking in Z-Dock software

| **Parameters** | **Value** |
| --- | --- |
| Angular step size | 6 |
| Distance cut-off | 10.0 |
| ZRank | True |
| Zrank Top poses | 10 |
| Clustering Top poses | 10 |
| Clustering RMSD cut-off | 10.0 |
| Clustering Interface cut-off | 10.0 |
| Maximum number of clusters | 2 |
| Parallel processing | False |
| Parallel processing server order | True |
| Use electrostatic and desolvation energy | True |

Table S4. Physico-chemical property analysis of Pi4 orthologue proteins

| **Protein name** | **pI** | **GRAVY** | **Instability index** | **Aliphatic index** |
| --- | --- | --- | --- | --- |
| Pi4of | 0.16 | -0.026 | 0.89 | 108.02 |
| Pi4 | 0.00 | -0.04 | 8.26 | 104.00 |
| Pi4rh | 0.09 | -0.03 | 3.02 | 104.0 |
| Pi4tp | 0.18 | 0.062 | 6.94 | 106.9 |
